# The archaeo-eukaryotic GINS proteins and the archaeal primase catalytic subunit PriS share a common domain

**DOI:** 10.1186/1745-6150-5-17

**Published:** 2010-04-12

**Authors:** Agnieszka Swiatek, Stuart A MacNeill

**Affiliations:** 1 Centre for Biomolecular Sciences, School of Biology, University of St Andrews, North Haugh, St Andrews, Fife KY16 9ST, UK

## Abstract

Primase and GINS are essential factors for chromosomal DNA replication in eukaryotic and archaeal cells. Here we describe a previously undetected relationship between the C-terminal domain of the catalytic subunit (PriS) of archaeal primase and the B-domains of the archaeo-eukaryotic GINS proteins in the form of a conserved structural domain comprising a three-stranded antiparallel β-sheet adjacent to an α-helix and a two-stranded β-sheet or hairpin. The presence of a shared domain in archaeal PriS and GINS proteins, the genes for which are often found adjacent on the chromosome, suggests simple mechanisms for the evolution of these proteins.

**Reviewers:**

This article was reviewed by Zvi Kelman (nominated by Michael Galperin) and Kira Makarova.

## Findings

Primases are specialised DNA-dependent RNA polymerase enzymes that function in chromosome replication to synthesise oligoribonucleotide primers for use by the replicative DNA polymerases [1,2]. Structurally, primases fall into two classes. One class comprises the DnaG family enzymes found in bacteria and archaea. The second class are the heterodimeric primases of the archaeo-eukaryotic primase (AEP) superfamily found in the eukarya and archaea but which are also present in some bacteria [3]. The AEP enzymes comprise a catalytic and a non-catalytic subunit. In the archaea these are designated PriS and PriL, respectively. In eukaryotes, the dimeric primase forms part of the replicative DNA polymerase α-primase complex that initiates Okazaki fragment synthesis.

The first structural insights into archaeal primase function came from the crystal structures of the PriS proteins from the euryarchaeal organisms *Pyrococcus furiosus *[4] and *P. horikoshii *[5]. The latter was co-crystallised with UTP (uridine-5'-triphosphate) allowing confirmation of the location of the active site of the enzyme. The *P. furiosus *and *P. horikoshii *PriS proteins are composed of two distinct domains: a mixed α/β domain (the Prim domain) that includes the catalytic site of the enzyme and a smaller α-helical domain of unknown function [4,5].

In addition to the *Pyrococcus *PriS structures, the structure of the PriS protein from the crenarchaeal organism *Sulfolobus solfataricus *has also been determined [6]. Three significant differences are apparent when comparing the *S. solfataricus *PriS structure with those of the *Pyrococcus *PriS proteins: the α-helical domain observed in the latter proteins is reduced to a single irregular helix in *S. solfataricus *PriS, the zinc binding motif in *S. solfataricus *PriS is located at the end of an extended β hairpin structure that is absent from the *Pyrococcus *proteins, and a mixed α/β domain of ~50 amino acids (termed the PriS-CTD) is found at the C-terminal end of the *S. solfataricus *protein but is also absent from the *Pyrococcus *proteins [6]. The PriS-CTD, which is the subject of this report, comprises a three-stranded antiparallel β-sheet adjacent to an α-helix and a two-stranded antiparallel β-sheet. Multiple sequence alignments (data not shown) indicate that the PriS CTD is conserved in all archaeal lineages with the exception of the *Thermococcales *(including *Pyrococcus *and *Thermococcus *species) and the *Methanobacteriales *(*Methanosphaera *and *Methanothermobacter *species), implying that these latter groups have undergone lineage-specific loss of this domain. In addition, the PriS-CTD does not appear to be present in the eukaryotic primase small subunit proteins. The role of the PriS-CTD is unclear but it has been suggested that this may play a role supporting and positioning the extended β hairpin structure that forms the stem of the zinc-binding motif [6]. In the *Pyrococcus *PriS proteins, which lack the extended β hairpin, a single α-helix replaces the PriS-CTD [4,5].

The function of the non-catalytic primase subunit is less clear but experiments suggest that this protein might have a role in determining (or limiting) the length of the RNA primer synthesised by the catalytic subunit [7]. Three-dimensional structures for truncated *S. solfataricus *and *P. horikoshii *PriL proteins have been determined and the PriS-PriL subunit interface defined [6,8]. Missing from both PriL structures is the C-terminal [4Fe-4S] cluster-containing domain that is found conserved in the eukaryotic non-catalytic primase subunit and which has been shown to be essential for primer synthesis [9,10].

DNA unwinding during eukaryotic chromosome replication is most likely catalysed by the CMG (Cdc45-MCM-GINS) complex comprising the hexameric MCM DNA helicase and its accessory factors, the Cdc45 protein and GINS [11,12]. Eukaryotic GINS is a heterotetramer consisting of the Sld5, Psf1, Psf2 and Psf3 subunits, each of which comprises two distinct protein domains [13,14]: an A-domain composed largely of α-helices and a smaller B-domain made up largely of β-strands [15-17]. Intriguingly, the order of the two domains is circularly permuted in the Sld5 and Psf1 subunits compared to the Psf2 and Psf3 subunits [18,19]. In Sld5 and Psf1 the A-domain is N-terminal to the B-domain, whereas in Psf2 and Psf3 it is the B-domain that is N-terminal. In the complex, the four subunits of GINS are arranged in two layers and the B-domains appear to function both to stabilise the interfaces between the layers of the complex and to mediate protein-protein interactions with additional factors [15-17]. The broader function of GINS within the CMG complex is not known and although several models have been proposed, significant uncertainty remains over the mode of action of the MCM helicase itself [13,14]. It has been suggested, for example, that MCM acts primarily as a double-stranded DNA translocase, pumping dsDNA through its central cavity in an ATP-dependent manner; DNA exiting the central channel might then encounter the GINS protein acting as a ploughshare to sterically separate the two DNA strands [20]. Further biochemical analysis of CMG function will be required to resolve this uncertainty.

All archaeal genomes sequenced to date encode a single protein with similarity to the eukaryotic Sld5 and Psf1 proteins and their characteristic A-B domain order [18,19]. A subset of species, including representatives of the deeply-branching *Thaumarchaeota *[21] and *Korarchaeota *[22], encode an additional protein (called Gins23) with similarity to the eukaryotic Psf2 and Psf3 proteins and their B-A domain order. In *S. solfataricus *and *P. furiosus*, the Gins51 and Gins23 proteins form a tetrameric complex comprising two molecules of Gins51 and two of Gins23 that is likely to be similar in structure to eukaryotic GINS [18,23]. The structure of the GINS complex in those archaea that apparently lack Gins23 is not known; in particular, it is not known if the Gins51 protein can form tetramers. In evolutionary terms, it is likely that the last common archaeo-eukaryotic ancestor encoded proteins with both A-B (Gins51) and B-A (Gins23) domain order [18,19]. In eukaryotic cells, subsequent duplication of the ancestral genes encoding Gins51 and Gins23 produced Sld5 and Psf1, and Psf2 and Psf3, respectively, while in the archaea, lineage specific loss of the gene encoding Gins23 led to the appearance of species lacking this protein [18,19].

In the course of database searching to identify GINS proteins in diverse archaeal species, we observed that sequences corresponding to the C-terminal domain (CTD) of the catalytic subunit of the archaeal primase protein PriS were often detected when archaeal GINS proteins were used as the query sequence. For example, BLAST searching using default parameters [24] against archaeal proteins in the NCBI Reference Sequence database [25] with the *Cenarchaeum symbiosum *(strain A) Gins23 protein [CENSYa_1724; GI: 118576897] as the query identifies the PriS protein [PAE3036; GI: 1463797] from *Pyrobaculum aerophilum *with an E-value 0.003 (amino acids 14-63 of the *C. symbiosum *Gins23 are 42% identical to residues 261-310 of *P. aerophilum *PriS). Additional *Pyrobaculum *PriS proteins, from *P. calidifontis *[Pcal_0991, GI: 4909914]. *P. arsenaticum *[Pars_1787, GI:5055591] and *P. islandicum *[Pisl_0437, GI: 4617745], are found with E-values of 0.058, 0.063 and 0.22, while PriS from *Thermoproteus neutrophilus *[Tneu 1683; GI: 6165219] is found with an E-value of 5.6.

**Figure 1:**
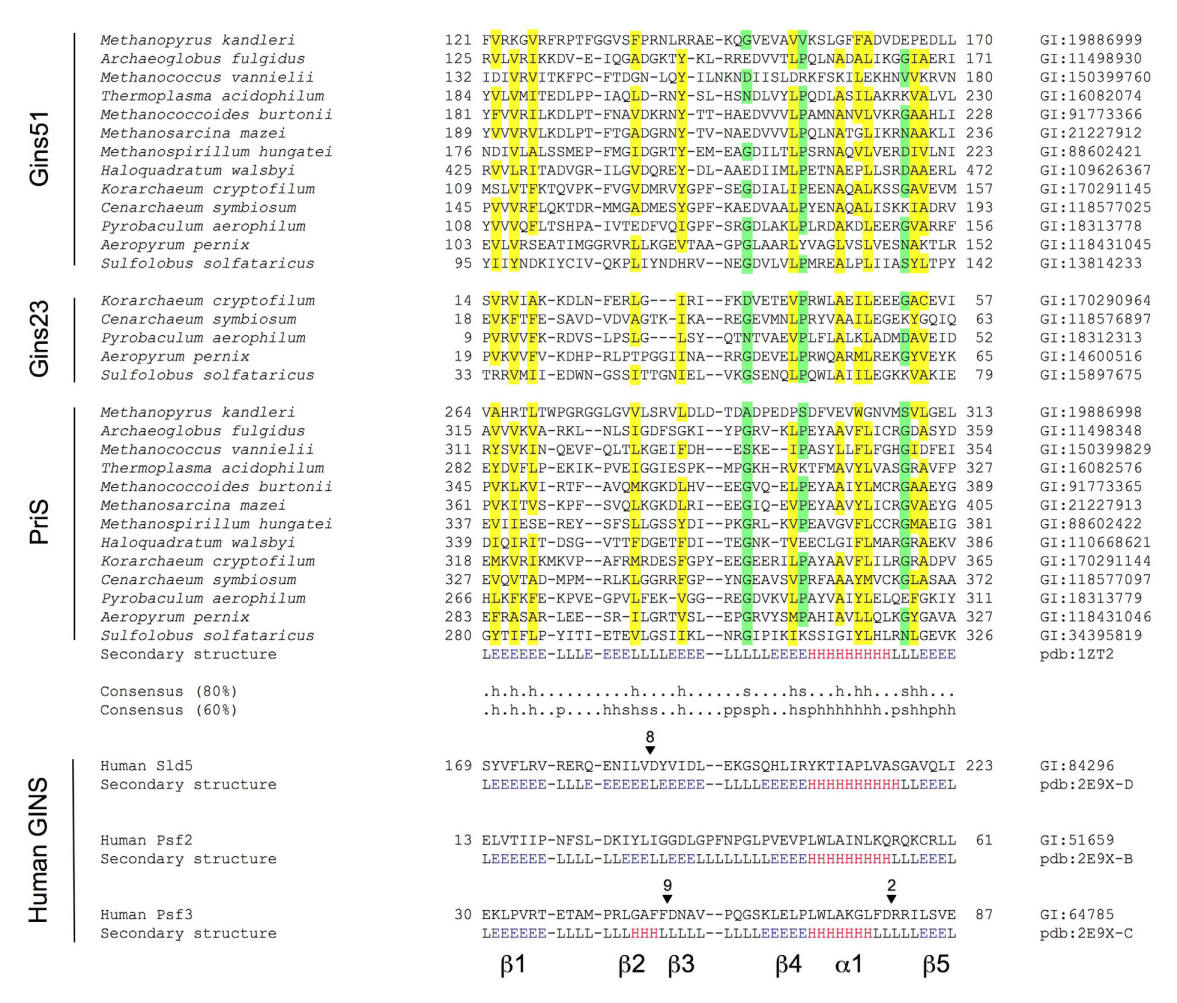
**Multiple sequence alignment of archaeal primase CTDs and archaeal and eukaryotic GINS B-domains**. The multiple sequence alignment of PriS CTD and archaeal GINS B-domains was generated using Clustal X 2.0 [28,29] with default parameters. Sequences are denoted by their species names (left) and numeric Genbank Identifiers (GI numbers, right). The positions of the first and last residues of the aligned region of the corresponding protein are indicated. The colouring is based on the consensus shown underneath the alignment. Hydrophobic positions (ACFILMVWYH) are indicated by the letter h and shaded yellow when present in 80% of the sequences shown; small residues (ACDGNPSTV) are indicated by the letter s and shaded green. The secondary structure of the CTD of the *S. solfataricus *PriS protein (PDB code 1TZ2) is shown underneath the alignment (with H, E and L indicating α-helix, β-strand and loop regions respectively, with α-helices shown in red and β-strands in blue), as are the primary sequences and secondary structures of the B-domains of three of the four human GINS proteins: Sld5, Psf2 and Psf3 (derived from PDB file 2E9X). The alignment of the human GINS and *S. solfataricus *PriS CTD sequences was generated by pairwise structure comparison (1ZT2 versus 2E9X with default parameters) using DaliLite [27]. The inverted triangles above the Sld5 and Psf3 sequences indicate that amino acids have been omitted at these positions; the number of amino acids omitted is shown.

**Figure 2:**
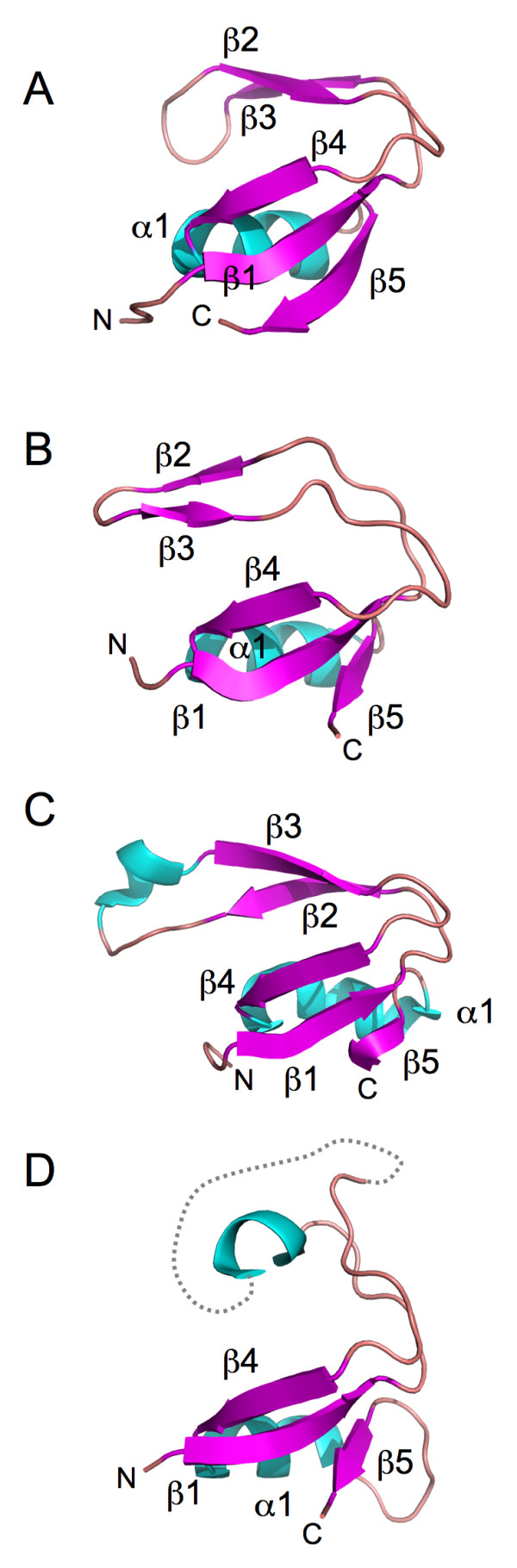
**Primase CTD and GINS B-domain structures share a common fold**. **A**. Structure of the C-terminal domain (CTD, amino acids 274-329) of the *S. solfataricus *PriS protein (PDB code 1ZT2, chain A) with the five conserved β-strands β1-β5 and helix α1 indicated. **B**. Structure of the N-terminal B-domain (amino acids 12-61) of the human GINS subunit Psf2 (PDB code 2E9X, chain B). **C**. Structure of the C-terminal B-domain (amino acids 167-223) of human Sld5 (PDB code 2E9X, chain D). Note the presence of an additional α-helix between β-strands β2 and β3. **D**. Structure of the N-terminal B-domain (amino acids 30-87) of the human Psf3 (PDB code 2E9X, chain C). Amino acids 48-56 are missing from the structure (indicated by broken line).

While not providing unambiguous evidence of relatedness, these results prompted us to explore possible relationships between the PriS CTD and the GINS B-domain in greater detail. Figure 1 shows a multiple sequence alignment of PriS CTD and GINS B-domains from a representative set of archaeal species, revealing low-level sequence conservation across the entire CTD and B-domain regions (see also Additional file 1). To ask whether this apparent sequence conservation was indicative of structural similarity between the PriS CTD and GINS B-domains, we compared the three-dimensional structures of *S. solfataricus *primase (PDB 1ZT2) [6] with the human GINS structure (PDB 2E9X) [15-17] using DaliLite [26,27]. Structural similarities between the PriS CTD and B-domains of Sld5, Psf2 and Psf3 were readily identified, with Z-scores of 5.6, 5.5 and 3.4 and rmsd values of 2.6, 2.6 and 1.9 Å for 50, 48 and 38 Cα atoms, respectively (Figure 1). As noted above, the PriS CTD comprises a three-stranded (β1, β4, β5) antiparallel β-sheet adjacent to an α-helix and a two-stranded (β2, β3) antiparallel β-sheet (Figures 1 and 2A). The structural similarity is most apparent with the Psf2 B-domain, which also comprises five β-strands and a single α-helix (Figures 1 and 2B). As with the PriS CTD, strands β1, β4 and β5 interact with the α-helix, while strands β2 and β3 form a hairpin-like structure. The B-domain of Sld5 forms a similar structure, albeit with an extra α-helix located between β2 and β3 (Figures 1 and 2C), while the B-domain of Psf3 is more diverged (and partly invisible in the crystal structure) but the three-stranded β-sheet and α-helix are still present (Figures 1 and 2D). The structure of the B-domain of the human Psf1 protein is not known nor have any of the archaeal GINS proteins been crystallised.

**Figure 3:**
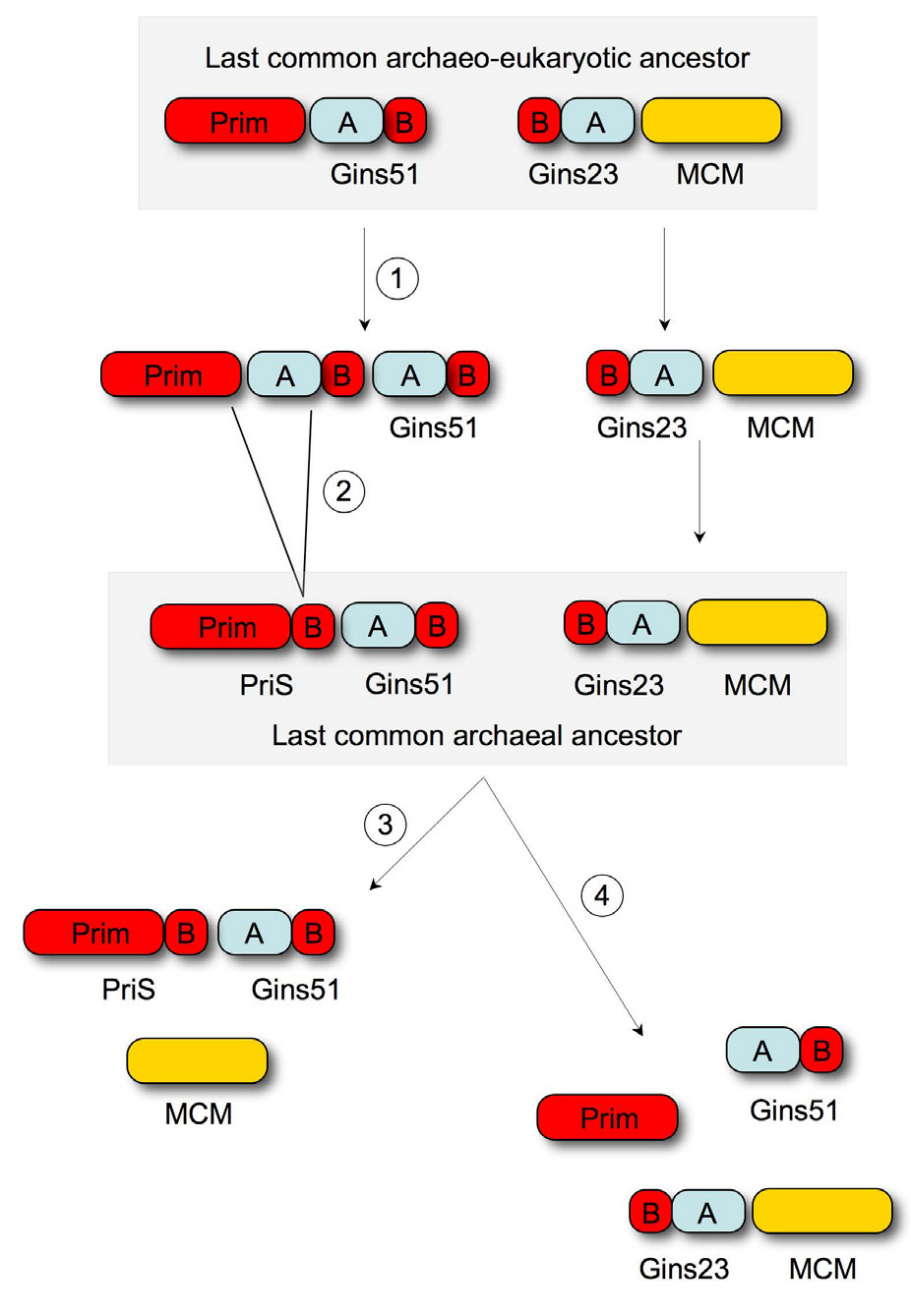
**Model for acquisition of the CTD by PriS**. Tandem duplication (labelled 1) of a Gins51 ORF found adjacent to a Prim domain ORF in the last common archaeo-eukaryotic ancestor is followed by deletion (labelled 2) of Gins51 A-domain sequences resulting in fusion of Prim domain and B-domain sequences and creation of an ORF encoding a recognisable PriS protein in the last common archaeal ancestor. Subsequent archaeal evolution has seen loss of Gins23 (labelled 3) in many species and loss of the CTD (labelled 4) from PriS in the *Thermococcales*, including *Pyrococcus *and *Thermococcus *species, and the *Methanobacteriales*. Co-localisation and co-expression of ORFs is also absent in many extant species [13].

The finding that the PriS CTD and GINS B-domains are related to one another may shed light on the evolution of these proteins. In many archaeal species, the ORFs encoding PriS and Gins51 are adjacent to one another on the chromosome and in certain cases, overlap [13,18,19]. This arrangement is seen in the *Korarchaeota*, for example, a deeply branching archaeal clade [22]. The close physical proximity of the PriS and Gins51 ORFs suggests a simple mechanism for the acquisition of the CTD by PriS by way of straightforward sequence duplication and deletion. In the model shown in Figure 3, the last common archaeo-eukaryotic ancestor is proposed to encode a Prim domain and Gins51 from one pair of adjacent ORFs and Gins23 and MCM from second pair of adjacent ORFs (Figure 3). This reflects the physical organisation of PriS, Gins51, Gins23 and MCM genes in several highly-diverged extant archaeal genomes. Following a tandem duplication of Gins51 (labelled 1 in Figure 3), deletion of sequences between the Prim domain and B-domain of the immediately adjacent Gins51 ORF (labelled 2) results in formation of a PriS protein complete with CTD as a Prim-B-domain fusion. The fact that the eukaryotic primase small subunit lacks the CTD may imply that these duplication and fusion events took place after the divergence of the eukaryotic and archaeal lineages. Thus, the last common archaeal ancestor encodes PriS adjacent to Gins51 and Gins23 adjacent to MCM. During subsequent archaeal evolution, Gins23 has been lost from many lineages (labelled 3 in Figure 3) and the CTD lost from the *Thermococcales *and *Methanobacteriales*, including *Pyrococcus *species (labelled 4). Co-localisation of the ORFs is also lost in many extant species [13].

In conclusion, the observations described here highlight a previously undetected relationship between two key components of the archaeal replication machinery and suggest a simple mechanism to account for the evolution of the PriS protein.

## List of abbreviations

BLAST: basic local alignment search tool; CTD: C-terminal domain; PSI-BLAST: basic local alignment search tool; GINS: go-ichi-ni-san; CMG: Cdc45-MCM-GINS; ORF: open reading frame.

## Competing interests

The authors declare that they have no competing interests.

## Authors' contributions

AS performed database searches, collated information, generated the multiple sequence alignments and aided in preparation of the manuscript. SM made the initial observations, coordinated the project and drafted the manuscript. Both authors read and approved the final manuscript.

## Reviewers' comments

### Reviewer's report 1

Zvi Kelman, University of Maryland Biotechnology Institute (nominated by Michael Galperin, National Center for Biotechnology Information)

The manuscript by Swiatek and MacNeill describes a structural comparison between domains of the eukaryotic GINS and the archaeal primase. It was found that although the domains share limited sequence similarities, they have similar three-dimensional folds. Using these observations, the authors proposed several mechanisms involving gene duplication that could result in the two protein families. These are interesting observations regarding essential replication enzymes in archaea and eukarya. I have only two minor comments. It would be useful for readers who are not familiar with the archaeal replication system to briefly describe the dimeric archaeal primase and the role of each subunit. A sentence or two regarding the proposed function(s) of the GINS complex would also be useful (in addition to the references provided).

***Authors' response: ****We are grateful for the reviewer's suggestions and have modified the text of the manuscript accordingly*.

### Reviewer's report 2

Kira Makarova, National Center for Biotechnology Information

Swiatek and MacNeill have made an interesting observation about the similarity of archaeal small primase subunit (PriS) C-terminal domain and B-domain of GINS-like proteins and have presented a plausible evolutionary scenario showing how the fusion of ancestral PriS and B-domain of GINS (specifically Gins51) could have occurred. This paper definitely extends the horizons of our understanding of complex events in the evolution of the molecular machinery for DNA replication initiation in archaea and eukaryotes. Importantly, it also provokes further discussion and analysis of the proteins and domains involved in this process. Specifically the absence of CTD in PriS in *Thermococcales *and *Methanobacteriales *and especially eukaryotes raises further questions about the actual ancestral state and involvement of horizontal transfer in the chain of evolutionary events. In this respect it would be interesting to see a phylogenetic tree reconstructed for Prim domain of PriS (of archaea and eukaryotes). While the evolutionary scenario suggested in this paper is really tempting because of physical proximity of PriS and Gins51 in some archaea, the ancestral state of this gene arrangement is not certain, since many archaea do not have it, including *Thaumarchaea*, one of deeply branching groups. Moreover the suggested scenario does not seem to take into account the observation that the CTD of archaeal PriS is a little bit more similar to B-domain of Gins23 (this follows from such data reported in the paper as PSI-BLAST search results and multiple alignment which shows that only eukaryotic Psf2 has structure and sequence fully compatible with the CTD while Sld5 has a specific insertion between β2 and β3). Hopefully structures of archaeal Gins51 and Gins23 would help to resolve some of these issues. Thus I would not be surprised if the evolutionary scenario of PriS/GINS evolution will be revised when new data became available. And, of course, many questions still remain about the configuration of the molecular complex that includes PriS and a variety of GINS proteins or/and CTD.

*Authors' response: *

*The reviewer is correct to state that the organisation of the genes encoding the PriS and Gins51 proteins in the last common archaeal ancestor is not certain and to hint that the lack of physical proximity between these genes in the deeply-branching Thaumarchaeota might be an indication that the Prim-Gins51 gene organisation proposed in our model for the acquisition of the CTD by PriS *(Figure 3) *is problematic, despite the widespread co-localisation of these genes in many diverse archaeal species including representatives of the Euryarchaeota, Korarchaeota and Crenarchaeota *(see Additional file 1). *The sequencing of additional archaeal genomes, particularly from the deeply-branching clades, will be of great importance in clarifying this issue*.

*In addition, while it is true that the findings reported here could be construed as suggesting a closer relationship between the PriS CTD and the B-domains of the Gins23 family proteins (Psf2 and Psf3 in eukaryotes), the low levels of sequence similarity displayed by the CTD and B-domains *(Figure 1) *and the substantial evolutionary distance between the archaeal and human proteins whose structures have been solved *(Figure 2) *do not allow firm conclusions to be drawn on this point. It also seems unlikely on the basis of the sequence alignment shown in *Figure 1* that the sequence insertion in the human Sld5 will be present in archaeal Gins51 B-domain. As the reviewer rightly points out, representative structures of archaeal Gins51 and Gins23 proteins may well help to resolve this issue*.

## Supplementary Material

Additional file 1 **Supplementary information**. Archaeal PriS, Gins51 and Gins23 proteins: accession numbers and operon organisation. Click here for file
