# Supplementary material for: The archaeo-eukaryotic GINS proteins and the archaeal primase catalytic subunit PriS share a common domain
Source: Biol Direct. 2010 Apr 12;5:17. doi: 10.1186/1745-6150-5-17 (PMC2861644; doi:10.1186/1745-6150-5-17)
Supplement: Additional file 1 — Supplementary information. Archaeal PriS, Gins51 and Gins23 proteins: accession numbers and operon organisation. [file 1745-6150-5-17-S1.PDF]

**The archaeo-eukaryotic GINS proteins and the archaeal primase catalytic subunit PriS share a common domain**

Agnieszka Swiatek and Stuart A MacNeill

**Supplementary information:**

Archaeal PriS, Gins51 and Gins23 proteins: accession numbers and operon organisation

| ***Species*** | ***Taxonomic group*** | ***PriS*** | ***Gins51*** | ***Gins23*** |
|---|---|---|---|---|
| *Cenarchaeum symbiosum* | *Thaumarchaeota* | GI:118577097 | GI:118577025 | GI:118576897 |
| *Korarchaeum cryptofilum* | *Korarchaeota* | GI:170291144 | GI:170291145 | GI:170290964 |
| *Pyrobaculum aerophilum* | *Crenarchaeota > Thermoproteales* | GI:18313779 | GI:18313778 | GI:18312313 |
| *Sulfolobus solfataricus* | *Crenarchaeota > Sulfobales* | GI:34395819 | GI:13814233 | GI:15897675 |
| *Aeropyrum pernix* | *Crenarchaeota > Desulfurococcales* | GI:118431046 | GI:118431045 | GI:14600516 |
| *Methanopyrus kandleri* | *Euryarchaeota > Methanopyrales* | GI:19886998 | GI:19886999 | - |
| *Methanococcus vannielii* | Euryarchaeota > *Methanococcales* | GI:150399829 | GI:150399760 | - |
| *Thermoplasma acidophilum* | *Euryarchaeota > Thermoplasmatales* | GI:16082576 | GI:16082074 | - |
| *Methanococcoides burtonii* | *Euryarchaeota > Methanosarcinales* | GI:91773365 | GI:91773366 | - |
| *Methanosarcina mazei* | *Euryarchaeota > Methanosarcinales* | GI:21227913 | GI:21227912 | - |
| *Methanospirillum hungatei* | *Euryarchaeota > Methanomicrobiales* | GI:88602422 | GI:88602421 | - |
| *Haloquadratum walsbyi* | *Euryarchaeota > Halobacteriales* | GI:110668621 | GI:109626367 | - |
| *Archaeoglobus fulgidus* | *Euryarchaeota > Archaeoglobales* | GI:11498348 | GI:11498930 | - |
| *Pyrococcus furiosus* | Euryarchaeota > *Thermococcales* | GI: 18976482 | GI: 18893033 | GI: 18892460 |
| *Methanosphaera stadtmanae* | Euryarchaeota > *Methanobacteriales* | GI: 84488915 | GI: 84490308 | GI: 146304792 |

Key: PriS and Gins51 are immediately adjacent on the chromosome (yellow shading), Gins23 and MCM are immediately adjacent on the chromosome (pale green shading); no co-localisation of PriS and Gins51 (pink shading); no co-localisation of Gins23 and MCM (orange shading). Species shaded green encode PriS lacking the CTD.
